# Phase delaying the human circadian clock with a single light pulse and moderate delay of the sleep/dark episode: no influence of iris color

**DOI:** 10.1186/1740-3391-7-8

**Published:** 2009-07-17

**Authors:** Jillian L Canton, Mark R Smith, Ho-Sun Choi, Charmane I Eastman

**Affiliations:** 1 Biological Rhythms Research Laboratory, Department of Behavioral Sciences, Rush University Medical Center, Chicago, IL, USA; 2 Department of Ophthalmology, Rush University Medical Center, Chicago, IL, USA

## Abstract

**Background:**

Light exposure in the late evening and nighttime and a delay of the sleep/dark episode can phase delay the circadian clock. This study assessed the size of the phase delay produced by a single light pulse combined with a moderate delay of the sleep/dark episode for one day. Because iris color or race has been reported to influence light-induced melatonin suppression, and we have recently reported racial differences in free-running circadian period and circadian phase shifting in response to light pulses, we also tested for differences in the magnitude of the phase delay in subjects with blue and brown irises.

**Methods:**

Subjects (blue-eyed n = 7; brown eyed n = 6) maintained a regular sleep schedule for 1 week before coming to the laboratory for a baseline phase assessment, during which saliva was collected every 30 minutes to determine the time of the dim light melatonin onset (DLMO). Immediately following the baseline phase assessment, which ended 2 hours after baseline bedtime, subjects received a 2-hour bright light pulse (~4,000 lux). An 8-hour sleep episode followed the light pulse (i.e. was delayed 4 hours from baseline). A final phase assessment was conducted the subsequent night to determine the phase shift of the DLMO from the baseline to final phase assessment.

Phase delays of the DLMO were compared in subjects with blue and brown irises. Iris color was also quantified from photographs using the three dimensions of red-green-blue color axes, as well as a lightness scale. These variables were correlated with phase shift of the DLMO, with the hypothesis that subjects with lighter irises would have larger phase delays.

**Results:**

The average phase delay of the DLMO was -1.3 ± 0.6 h, with a maximum delay of ~2 hours, and was similar for subjects with blue and brown irises. There were no significant correlations between any of the iris color variables and the magnitude of the phase delay.

**Conclusion:**

A single 2-hour bright light pulse combined with a moderate delay of the sleep/dark episode delayed the circadian clock an average of ~1.5 hours. There was no evidence that iris color influenced the magnitude of the phase shift. Future studies are needed to replicate our findings that iris color does not impact the magnitude of light-induced circadian phase shifts, and that the previously reported differences may be due to race.

## Background

Light exposure can shift the human circadian clock in a phase-dependent manner. Light exposure during the evening hours or early in the habitual sleep episode produces phase delays, while light exposure late in the habitual sleep episode or morning hours produces phase advances [1-4]. The crossover point at which the phase shift in response to light exposure changes from delays to advances is estimated to occur near the body temperature minimum (Tmin) [1]. The rate at which the circadian clock can be shifted with light exposure is dependent on the spectral composition of the light source [5-8], the light level [9,10], and the duration and pattern of the light pulse(s) [11-14]. As the converse of light exposure, the timing or duration of the sleep/dark episode can also phase shift the circadian clock [15-19].

Many studies have measured the phase delay produced by bright light exposure administered over successive days (e.g. [20-23]). Some studies have also administered phase delaying light pulses on a single day. Understanding how much a single pulse of light can delay the circadian clock is important because practical constraints in the real world may limit the ability of individuals to adhere to several consecutive days of light treatment. Phase delays of up to one hour per day can be produced when a phase delaying light pulse is combined with awakening at the usual time the next morning [24-26]. However, holding wake time constant would likely constrain phase delays of the circadian clock because morning light exposure on the advance portion of the light PRC would oppose the delaying effect of the evening/nighttime light pulse. When a very long single light pulse is combined with 2 days of a large abrupt shift of the sleep episode, the circadian clock can be delayed as much as three hours [9,27,28]. Although these delays can be quite large, delaying the sleep episode that much is not practical for most individuals trying to phase shift their circadian rhythms at home. When a phase-delaying evening light pulse is used in the field, it may be most practical to combine it with a moderate delay of the sleep/dark episode (e.g. [29,30]). The first goal of this study was thus to measure the phase delay produced by a single 2-hour light pulse before bedtime combined with a moderate delay of the sleep episode.

A number of studies have shown large individual differences in the magnitude of the phase shift produced within the same protocol (e.g. [8,12,24,31,32]). Factors that may contribute to these individual differences include light exposure history, iris color and/or race. Light history has been shown to influence light-induced melatonin suppression [33,34], and these findings have recently been extended to circadian phase shifting [32,35]. One study found that light-eyed subjects had earlier sleep times and more "morningness" on a chronotype questionnaire [36], which could suggest that light-eyed subjects are more sensitive to the phase-advancing effects of morning light exposure. Light-induced melatonin suppression has been reported to be greater for light-eyed Caucasian than dark-eyed Asian subjects [37]. In this latter study it could not be determined whether iris color, race, or both accounted for the group differences. Differences in intraocular straylight as a result of iris color could influence non-image-forming responses such as melatonin suppression and circadian phase shifts. Intraocular straylight (light scattering) is the name for the phenomenon in which the retina receives light at locations that do not optically correspond to the direction light is coming from, but that nonetheless could trigger phototransduction. Individuals with lighter pigmented irises experience greater intraocular straylight [38], possibly because transmission of light through lighter pigmented irises is greater than through darker pigmented irises [39], and thus might be expected to have larger non-image-forming responses.

We have recently reported that Caucasian subjects had a longer endogenous circadian period (tau), relative to African American subjects [40]. We also reported preliminary evidence that Caucasians have larger light-induced phase delays, and smaller light-induced phase advances [40]. Whether iris color contributes to differences in circadian responses independent of race is not yet clearly established. The second goal of this study was to test whether phase delays differed between light and dark-eyed subjects.

## Methods

### Subjects

Fourteen subjects completed the study, but data from one subject could not be used because there was no discernable dim light melatonin onset (DLMO). Table 1 shows the demographics of the remaining subjects. The subjects were healthy, nonsmokers, had an average BMI of 25.3 kg/m^2^, and did not show extreme morningness-eveningness [41]. In order to increase the likelihood of observing a difference in circadian phase shifts based on iris color, only subjects with blue or brown irises were enrolled in the study. Iris color was a subjective determination by more than one research assistant during the screening process. All subjects were medication free, except for one female on oral contraceptives. Subjects habitually consumed less than 300 mg of caffeine and 2 alcoholic drinks per day, and were free from common drugs of abuse, confirmed by a urine drug test at the start of the study. Subjects were screened for past or current medical, psychiatric, and sleep disorders via a telephone interview, an in-person interview, and several additional questionnaires. Subjects were not color blind (Ishihara Color blindness test). Subjects had not traveled across more than three time zones in the one month prior to or worked a night shift three months prior to beginning the study. The study was conducted in February of 2009. The study protocol was approved by the Rush University Medical Center Institutional Review Board, and all subjects provided written informed consent before study participation commenced.

**Table 1:** Subject demographics by iris color.

	Blue Eyes	Brown Eyes
N	7	6

Male/Female	5/2	3/3

Age (mean ± SD)	25.2 ± 6.0	25.8 ± 5.4

Self-reported		4 Caucasian
Race or Ethnicity	7 Caucasian	1 Hispanic 1 Asian

Owl-Lark Score	52.7 ± 6.1	52.3 ± 10.5

### Design

**Figure 1:**
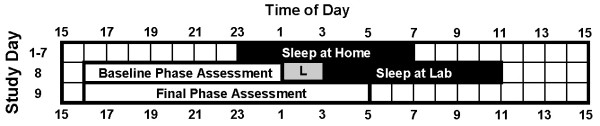
**Protocol diagram**. This protocol is for a subject sleeping from 23:00–7:00 at baseline (days 1–7). Shaded rectangle with L inside shows time of bright light exposure (2 h, ~4000 lux).

Figure 1 illustrates the protocol. During the baseline week (days 1–7), subjects were instructed to maintain a regular 8 hour sleep schedule each night. Their sleep schedule was similar to their habitual sleep schedule, as determined from pre-study sleep logs. Napping was prohibited. Subjects were required to call the lab voicemail within 10 minutes before bedtime and 10 minutes after wake time to verify compliance with the sleep schedule. Subjects completed daily event logs noting any caffeine, alcohol or over-the-counter medication/vitamins consumed that day. The day before the laboratory session (day 7), subjects were not allowed any caffeine or alcohol. On day 8, subjects arrived at the lab for a baseline phase assessment. At the end of the phase assessment, 2 hours after their habitual bedtime, subjects were exposed to bright light for 2 hours. After the light pulse, subjects slept in a private bedroom for 8 hours. Upon awakening, subjects remained in the lab bedrooms in <60 lux (4,100 K) and began their final phase assessment five hours later. After the laboratory session, photographs of subjects' irises were taken by an ophthalmologist (H.-S.C.). Photographs of subjects' left and right irises were taken using a Marco Ophthalmic slit lamp and Hitachi HV-D30 digital camera. Iris photographs were taken in the same dark room, with only the computer monitor and slit lamp for light. A 10 mm diameter circular straight beam of unfiltered light at 50% maximum brightness was used. Subjects were instructed to open their eyes wide, to expose the full iris.

### Bright Light Exposure

The bright light was administered via a single light box placed on a desk ~40 cm from the subjects' eyes. The light box (67 × 68 × 7 cm, Philips Lighting, Eindhoven, The Netherlands) contained four fluorescent 17,000K lamps. The spectral plots of these lamps have been published [42]. Subjects read during the light exposure. The targeted illuminance was ~4000 lux, irradiance ~1640 μW/cm^2^, and photon density ~4.2 × 10^15 ^of photons/cm^2^/sec. Every 20 minutes during the light treatment, research assistants measured the illuminance using a Minolta TL-1 illuminance meter to ensure that the light level was close to the targeted illuminance. To do this a research assistant told the subject to "freeze" in the position the subject was sitting, and then measured the light level from the subjects' head at the angle of gaze (which was typically downward since the subject was reading). If necessary, the research assistant then instructed the subject on how to reposition him/herself so that he/she were closer to the targeted light levels. If the subject was repositioned, the light level was measured again to verify that the light level was at the target level. When subjects were told to "freeze", the average light levels for the blue-eyed and brown eyed group were similar (3781 ± 286 lux vs 3521 ± 198 lux, respectively). After repositioning (which was not always necessary), light levels for the blue and brown-eyed subjects were 3806 ± 260 and 3901 ± 155 lux, respectively.

### Circadian Phase Assessments

The time of the phase assessments relative to subjects' baseline sleep schedules are shown in Fig 1. Details of phase assessment procedures have been previously described [43]. Subjects remained awake and recumbent in a dimly lit room (4,100 K lamps with red filters; < 5 lux; < 3.8 μW/cm^2^). Trips to an adjoining restroom, which was also maintained in < 5 lux, were permitted, but not in the 10 minutes preceding saliva samples. Every 30 minutes subjects provided a saliva sample using a salivette (Sarstedt, Newton, NC, USA). The samples were immediately centrifuged and frozen. At the end of both phase assessments, samples were shipped on dry ice to Pharmasan Labs (Osceola, WI) and radioimmunoassayed for melatonin. The sensitivity of the assay was 0.7 pg/ml and the intra- and inter-assay coefficients of variability were 12.1% and 13.2%, respectively.

### Data Analysis

To determine the DLMO, a threshold was calculated for each melatonin profile. The threshold was determined by calculating the mean of five consecutive low daytime melatonin values plus twice the standard deviation of these values [44]. Melatonin profiles were smoothed with a locally weighted least squares curve (GraphPad Prism, San Diego, CA). From each subject's two melatonin profiles (baseline and final), the higher of the two thresholds was applied to both profiles to calculate the DLMO. The DLMO was the point at which the fitted curve exceeded and remained above the threshold. Phase shifts were calculated by taking the difference between the baseline and final DLMO.

In order to quantify iris color, iris photographs were analyzed using color variables in Photoshop (Adobe Systems Incorporated, San Jose, CA). For each photograph, the iris was selected and the extraneous parts of the photo (i.e. pupil, eyelashes) were removed. Each iris photograph was quantified using two systems: RGB and LAB. The red-green-blue (RGB) system is a measure of the amount of red, green and blue hue present in an image. The three color dimensions of the RGB system yielded numbers ranging from 0–255, with smaller numbers indicating darker colors. The LAB system quantifies each image according to its lightness ("L") and its color axes ("A" and "B"). The lightness component of this system was determined for each iris photograph. For the lightness component, smaller numbers indicated darker irises, with a possible range of scores from 0 (black) to 100 (white). L and RGB values for the left and right irises of each subject were typically very similar, and were averaged for analyses.

Phase shifts of the DLMO for blue and brown-eyed subjects were compared with a two-tailed student's t-test. Pearson correlations were used to test the association between phase shift of the DLMO and iris color, as quantified with each of the RGB dimensions and the lightness component of the LAB system. Statistical significance was set at α = 0.05. Data are expressed as mean ± SD.

## Results

**Figure 2:**
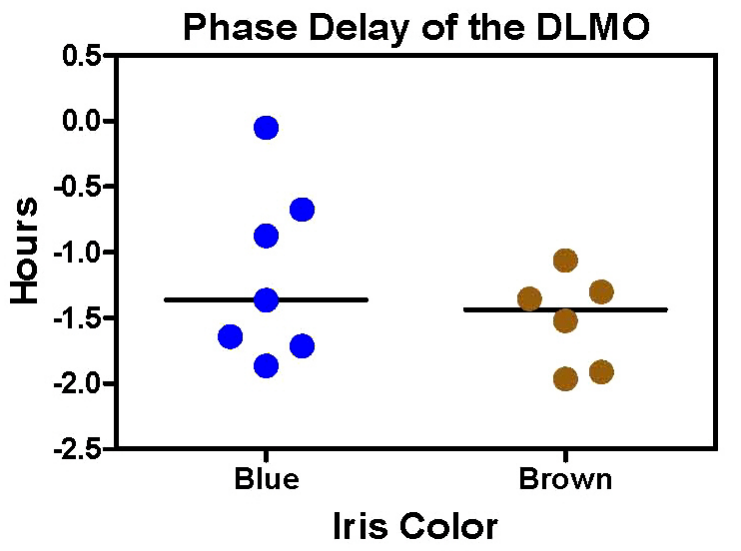
**Circadian rhythm phase delays with a single bright light pulse and delayed sleep/dark episode**. The horizontal lines represent the median phase delays.

The average phase delay of the DLMO was -1.3 ± 0.6 h, and the median phase delay was -1.4 h. There were large individual differences in phase shifts, with one subject delaying as little as 3 minutes, and others delaying about 2 hours (Fig 2). The average baseline DLMO was 22:22 ± 1.3 h, and the average baseline DLMO to baseline bedtime interval was 1.9 ± 1.3 h. The phase angle between the baseline DLMO to start time of the light pulse ranged from 2.3 to 6.5 h, and was similar for subjects with blue and brown irises. The correlation between this phase angle and phase shift of the DLMO was r = .51, p = 0.08, indicating a tendency for subjects receiving the light pulse closer to their DLMO to have larger phase delays.

There was no difference in the magnitude of the phase delay between subjects with brown (-1.5 ± 0.4) and blue (-1.2 ± 0.7) irises (Fig 2). As expected, quantification of iris color using the RGB and L metrics showed statistically significant differences between the blue and brown-eyed subjects for all variables except RGB Red (Table 2). However, there were no significant correlations between any of these variables and the phase delay of the DLMO (Table 2).

**Table 2:** Color dimensions (mean ± SD) for subjects with blue and brown irises.

Iris Color	Blue Irises	Brown Irises	Correlation with
Dimension	n = 7	n = 6	phase shift (n = 13)^b^
Blue^a^	125.5 ± 9.6**	42.7 ± 9.3	r = .40, p = 0.17

Green^a^	143.0 ± 5.2**	93.3 ± 18.0	r = .31, p = 0.31

Red^a^	130.1 ± 2.2	123.0 ± 17.3	r = .14, p = 0.64

Lightness	57.9 ± 1.4*	43.0 ± 7.2	r = .29, p = 0.33

* p < 0.01; ** p < 0.001, t-test comparing subjects with blue vs brown irises

^a ^Measured from 3 dimensions of the RGB color system.

^b ^Positive correlations indicate that subjects with darker irises had larger phase delays.

## Discussion

A single 2 hour bright light pulse at night combined with a 4 hour delay of the sleep/dark episode delayed the human circadian clock an average of ~1.5 hours. We also observed individual differences in the magnitude of the phase delay, from virtually no delay to up to 2 hours. These findings more clearly delineate the rate at which the circadian clock can be delayed in a practical protocol that could be used in the real world. Previous studies utilizing a single bright light pulse ending late at night with subjects waking at their habitual time (sleep episode truncated) have reported phase delays of about 1 hour [24,25]. Studies in which a single long duration bright light pulse (> 6 hours, up to ~10,000 lux) was paired with 2 days of a large delay (>8 hours) in the sleep/dark episode have reported phase delays of up to 3 hours [9,12,27,28]. Although a 3 hour phase delay from a single day of light treatment is robust, such a large shift in the sleep schedule may not be appealing or feasible for individuals using light treatment at home. The present study, which incorporated a compromise between not delaying wake time at all and completely inverting the sleep/dark episode, yielded large phase delays in a protocol that is more practical for real world applications.

We found that phase delays of the DLMO for subjects with blue and brown irises were similar. Light exposure measurements while subjects were sitting in front of the light box were not different between subjects with blue versus brown irises, and were close to the targeted light levels. The light levels in the 3 subjects in the blue-eyed group that had smallest phase delays were still at or close to the targeted light levels, suggesting that variability in the light levels reaching the cornea did not account for the variability in phase shifts of the DLMO. Caucasian subjects in the Higuchi et al. melatonin suppression study [37] had iris colors including blue, green and light brown, while all the Asian subjects had dark brown irises. We only enrolled subjects with blue or brown irises, but we could not clearly differentiate between subjects with light versus dark brown irises by visual inspection because there were continuous gradations in iris color. We therefore quantified iris color using individual color axes and lightness scales derived from each subject's iris photographs. Although the blue and brown-eyed groups were distinguished by several color dimensions derived from the iris photographs, none of these dimensions were associated with phase shift of the DLMO, or were even in the predicted direction. It is nonetheless possible that differences in phase shifting due to iris color might have been observed at lower light levels or with a different light source than used in the present study. It is also possible that, via disparate mechanisms, iris color influences light-induced melatonin suppression, but not circadian phase shifting.

The greater light-induced melatonin suppression in light-eyed Caucasians compared to dark-eyed Asians reported by Higuchi et al. [37] could have been due to race or iris color, since the two were confounded in their sample. We recently reported that African Americans subjects had a shorter tau than Caucasians [40]. In that manuscript we also re-analyzed data from our previous phase-advancing study with daily light pulses [42] that included both light and dark-eyed Caucasians as well as dark-eyed African Americans, and thus in which race and iris color were not completely confounded. We found that circadian phase advances in light (n = 6) and dark-eyed (n = 15) subjects were similar, but African Americans (n = 7) had larger phase advances than Caucasians (n = 11) [40], suggesting that race, not iris color, was a factor mediating the magnitude of circadian phase shifts.

Although there are racial differences in retinal anatomy, we hypothesize that the racial differences in phase shifting [40] are not due to racial differences in retinal anatomy or function, but rather are due to racial differences in tau. African Americans have darker fundus [45,46], likely due to greater choroidal melanin levels [47]. These anatomical differences could suggest that African Americans would have smaller phase shifts, since the darker fundus and higher melanin levels would absorb more light, and reduce the amount of light reflected from the outer retina that could potentially trigger phototransduction. Contrary to that suggestion, in our previous study [40] African Americans had *larger *phase advances than Caucasians. Because we found that African Americans had a shorter tau than Caucasians, which would augment phase advances relative to the Caucasians with a longer tau, we hypothesize that this larger phase advance in African Americans was due to differences in tau rather than differences in ocular structure.

One limitation of this study, which is a possible source of variability in these data, is that we did not measure light exposure history, which influences the magnitude of subsequent light-induced phase delays [32]. Similar large individual differences have been reported in other phase shifting studies (e.g. [8,12,24,32]), some of which either controlled for or measured light exposure history. It is theoretically also possible that light exposure history was systematically different for subjects with blue or brown irises, such that one group was exposed to more light than the other group, thereby confounding the group differences in the magnitude of the phase delay. A further limitation of this study is the relatively small sample size, since small differences in the magnitude or the variability of phase shifts between subjects with different iris colors might be observed with the greater statistical power that a larger sample size provides. A final note about this study is that we did not measure pupil size, and it is possible that subjects with blue irises had more pupil constriction than subjects with brown irises, diminishing the difference of retinal irradiance between the groups. However, because the light levels used in our study were above those that elicit a maximal pupil constriction in humans [48], and we think it is unlikely that pupil diameter contributed substantially to our results.

## Conclusion

With a single day of a 2-hour bright light pulse at night and a 4-hour delay of the sleep episode, the human circadian clock can be delayed an average of ~1.5 hours. This is a larger delay than studies that have administered a single phase delaying bright light pulse combined while maintaining habitual wake time. There were no differences in the phase delay between subjects with blue versus brown irises, and no association between objective measures of iris color or lightness/darkness and the magnitude of the circadian phase delay. Therefore, there was no evidence that iris color influenced the circadian phase delays produced by nighttime bright light exposure and a moderate delay of the sleep episode. Future studies could confirm that iris color does not, and racial differences do, influence the magnitude of light-induced circadian phase shifts.

## Competing interests

The authors declare that they have no competing interests.

## Authors' contributions

JLC helped design the study, supervised staff and subjects, screened and ran participants, performed data analyses, and prepared figures. MRS conceived the study, helped design the study, wrote the subject informed consent document, and commented on data analyses. H-SC performed iris photography. CIE helped design the study, was principal investigator on the grant supporting this research, and commented on data analyses. Each author contributed to manuscript composition and approved the final manuscript.
